# Multiple Batches of Fermentation Promote the Formation of Functional Microbiota in Chinese Miscellaneous-Flavor Baijiu Fermentation

**DOI:** 10.3389/fmicb.2020.00075

**Published:** 2020-01-31

**Authors:** Pulin Liu, Lihong Miao

**Affiliations:** College of Biological and Pharmaceutical Engineering, Wuhan Polytechnic University, Wuhan, China

**Keywords:** multiple batch, baijiu, functional microbiota, high-throughput sequencing, fermentation parameters

## Abstract

Baiyunbian baijiu, the most popular miscellaneous-flavor baijiu in China, has been widely consumed for decades. Similar to many Chinese baijiu, Baiyunbian baijiu is fermented in five successive batches every year. Sensory analysis demonstrated that the raw baijiu obtained from the last two fermentation batches always has better quality than that produced from the former three batches. In this study, the microbial compositions of fungi and bacteria in each fermentation batch were investigated via high-throughput sequencing. The results showed that *Bacillus*, *Virgibacillus*, and *Lactobacillus* dominated the bacterial community in the last two batches, and the most prevalent fungi were *Paecilomyces*, *Saccharomyces*, and *Zygosaccharomyces*. In contrast, large percentages of fungi belonging to *Thermomyces*, *Thermoascus*, *Monascus*, and *Issatchenkia* and prokaryotes belonging to *Acetobacter*, *Lactobacillus*, and *Thermoactinomyces* were observed in the former three fermentation batches. GC-MS analysis revealed that the fermented grains sampled from the latter two batches contained high concentrations of ethyl lactate, 2,3-butanediol and ethyl caproate, which were mainly generated by co-fermentation of *Lactobacillus* and yeast. The high acidity of the fermented grains in the fourth and fifth fermentation batches as well as the large contents of ethanol and moisture promoted the formation of the functional microbial community. This study provides insight into factors that influenced the baijiu fermentation and is helpful for developing new fermentation techniques with higher baijiu quality.

## Introduction

In human history, many foods are processed and preserved by spontaneous fermentation. Products obtained through successful spontaneous fermentation often exhibit special characteristics associated with their aroma, flavor, or nutritional properties (Navarrete-Bolaños, 2012). Chinese baijiu, regardless if it is an expensive brand or homemade brewage, is mainly fermented by traditional methods; no microorganisms are intentionally inoculated into the fermentation process (Li et al., 2011). Based on flavor characteristics, Chinese baijiu is traditionally divided into five categories, including soy sauce, light, strong, sweet honey and miscellaneous (Xiu et al., 2012). Baiyunbian baijiu is one of the most famous miscellaneous baijiu widely consumed in the central part of China (Xiong et al., 2014). The production of this baijiu can be classified into three parts in each fermentation batch, including starter fermentation, stacking fermentation, and alcohol fermentation in pits (Li et al., 2016). And then, the fermented grains are distilled, and the distillate is collected. This manufacture process is circulated five times each year. After the former fermentation batch is completed, new starter is inoculated to initiate the next batch of fermentation.

Fermenting multiple batches aims to obtain an optimal microbial composition dominated by functional microorganisms, including filamentous fungi, yeasts and bacteria (Zheng and Han, 2016). Filamentous fungi are dominant microorganisms in stacking fermentation, during which they produce enzymes to degrade the raw material into fermentable sugars (Chen et al., 2014). These sugars become substrates that are used for ethanol fermentation and volatile compound production by yeasts and bacteria. Although alcohol and water are the principal components of Chinese baijiu, the quality of the final product mainly depends on the concentration of other volatile compounds, such as esters, higher alcohols, acids, and phenols (Li et al., 2014). The generation of these volatile components is mainly influenced by the microbial succession during fermentation. Based on the olfactory test, raw Baiyunbian baijiu obtained from the fourth and fifth fermentation batches always present high quality than that distilled from the former batches every year (Liu, 2015). However, changes in the microbial community among different batches remains obscure. Wu et al. (2013) analyzed the yeast dynamics in four successive fermentation batches of Chinese soy sauce-flavor baijiu by using plate-spreading method. The results demonstrated that the yeast community of the first and second batches were similar, *Saccharomyces cerevisiae*, *Saccharomyces uvarum*, *Pichia membranifaciens*, *Torulaspora delbrueckii*, *Pichia anomala*, and *Saccharomyces servazzii* were identified. Two new species were detected in the third and fourth fermentation batches, including *Issatchenkia orientalis* and *Pichia scaptomyza*. In 2014, the total bacteria count was evaluated in Baiyunbian baijiu fermentation through a culture-dependent method. During stacking fermentation, the bacterial concentration slowly decreased from 1.24 × 10^7^ to 8.5 × 10^6^ CFU/g in the third batch and then increased to 7.45 × 10^7^ CFU/g in the end. Similar variations were observed in the alcohol fermentation stages (the total count declined from 9.5 × 10^4^ to 3.6 × 10^4^ CFU/g in the third batch and then increased to 6.5 × 10^4^ CFU/g in the fifth batch) (Xiong et al., 2016).

In this study, microbial communities in five successive Baiyunbian baijiu fermentation batches were characterized by high-throughput sequencing. Moreover, the chemical properties and volatile profiles (temperature, acidity, moisture, ethanol, reducing sugar, and starch content) of the fermented grains were detected to understand the correlation between the microorganism succession and the dynamics of fermentation factors. The results could help improve the baijiu fermentation techniques and baijiu quality.

## Materials and Methods

### Sample Collection

Stacking-fermented (48 h) and ethanol-fermented samples (15 days) were obtained from Baiyunbian Co., Ltd. (Jingzhou, China). Five successive fermentation batches were analyzed. The stacking-fermented samples were labeled as S1–S5, while ethanol-fermented samples obtained in pits were designated as L1–L5. At each sampling event, 20 subsamples were obtained and immediately homogenized. Approximately 200 g of the fermented grains were collected in triplicate to obtain adequate representation. The samples were transported into a laboratory at 4°C. Part of the fermented grains was used to extract DNA, and the remaining part was employed for analysis of physiochemical parameters to reveal the correlation between microbial community and environmental factors.

### Chemical Properties of Fermented Grains

Ten grams of fermented grains were mixed with 90 mL of distilled water and ultrasonically treated at 0°C for 30 min. The suspension was centrifuged at 2,000 *g* for 5 min, and the supernatant was pipetted to analyzed the contents of reducing sugar and ethanol. Reducing sugar was analyzed using DNS method (Miller, 1959). Ethanol content analysis was carried out on an Agilent 7820A GC system equipped with a DB-Wax column (30 m × 0.25 mm × 0.25 μm, J&W Scientific, CA, United States). The oven temperature was held at 60°C for 5 min and then raised to 230°C at a rate of 10°C min^–1^ before being held at 230°C for 5 min. The carrier gas was helium with a flow-rate of 1 mL min^–1^. Ethanol contents were quantified by comparing the peak areas with authentic standards. Water content in the fermented grains was measured by drying the samples at 105°C for 24 h. The sampling site temperatures were measured using a thermometer at the time of sampling. Acidity and starch content were assayed using the method described by Wu et al. (2013).

### DNA Extraction and High-Throughput Sequencing

For each replicate, 50 g of the fermented grains were suspended in 100 mL of DNA extraction buffer (40 mM EDTA, 50 mM Tris–HCl, pH8.0). The suspension was treated with lysozyme (1 mg/mL) and lyticase (2 mg/mL) at 37°C for 1 h. Genomic DNA was extracted using the method described by Li et al. (2011). For high-throughput sequencing, the bacterial 16S rRNA genes (V3–V4 region) and the fungal ITS1 region were amplified (Table 1). All DNA extraction and PCRs were carried out in triplicate. The resulting amplicons were separately sequenced by the Illumina Miseq platform (Illumina Corporation, San Diego, CA, United States).

**TABLE 1:** Primers used in this study.

Region	Primer	Sequence	References
V3–V4	341F	5′-CCTAYGGGRBGCASCAG-3′	Lee et al. (2017)
	806R	5′-GGACTACNNGGGTATCTAAT	
ITS1	1737F	5′-GGAAGTAAAAGTCGTAACAAGG-3′	Li et al. (2017)
	2043R	5′-GCTGCGTTCTTCATCGATGC-3′	

### Bioinformatics and Statistical Analysis

The raw sequencing reads were categorized by identifying sample tags, trimmed to improve the sequence quality and denoised using QIIME (Caporaso et al., 2010). Masked sequences with more than 50% of low complexity, sequences that contained more than two unresolved nucleotides and chimera sequences were removed. Operational taxonomic units (OTUs) were determined by a 3% cut-off. Maximal OTU richness was estimated using Michaelis–Menten models (*S_*chao*__1_*) (Hughes et al., 2001). OTU diversity and distribution were represented using Shannon diversity indices. For the taxonomic annotation, the consensus sequence for each OTU was queried by BLAST. Bacteria were searched on the Ribosomal Database Project II database (Cole et al., 2009), whereas yeasts or molds were identified on the UNITE database (Kõljalg et al., 2013). Clustering analysis was conducted with unweighted-pair group method using average linkages based on Bray–Curtis distance (Hammer et al., 2001). One-way analysis of variance (ANOVA) was performed (SPSS 20.0, IBM, United States) to determine the minimum significant difference (*p* < 0.05) in the biodiversity (Shannon index) and physiochemical properties of fermented grains. LSD-*t* test was used given that normal distribution (Shapiro–Wilk test) was observed in all groups. Microorganisms that led to similarities between the stacking or ethanol fermentation stage and the dissimilarity among different fermentation batches were analyzed using SIMPER (Clarke et al., 2014). Redundancy discriminant analysis (RDA) was performed using CANOCO 5.0 software (Šmilauer and Lepš, 2014).

### Analysis of Volatile Compounds by Using Headspace Solid-Phase Microextraction Coupled With GC-MS (HS-SPME-GC-MS)

Volatile compounds in fermented grains were measured by HS-SPME-GC-MS. The fermented grains were pretreated as follows. Five grams of the fermented grains were added to 20 mL of sterile water. The mixture was ultrasonically treated for 30 min at 4°C, and then centrifugated at 8,000 × *g* for 10 min. Eight milliliter of the supernatant and 10 μL of the internal standard (2-methyl-2-butanol, pentyl acetate, and 2-ethyl butanoic acid) were pipetted into a 20 mL vial with 3.0 g of NaCl. The sealed samples were equilibrated at 45°C for 10 min and extracted with SPME device for 45 min. GC-MS analysis was performed on Agilent 7820A GC system equipped with a DB-Wax column as described by Kong et al. (2014).

## Results

### Physiochemical Properties of Fermented Grains

Table 2 lists the physiological and chemical properties of the fermented grains. Each fermentation batch of Baiyunbian baijiu constituted with a stacking fermentation with a high temperature (40.0–53.5°C) and an alcohol fermentation stage with a low temperature (39.1–47.1°C). The temperature of the fermented grains depends mainly on microorganism growth, which is an important parameter that determines the development of ethanol production. Ethanol was mainly produced in the alcohol fermentation stage, and only a small amount of ethanol was generated in the stacking fermentation stage. The ethanol contents in L3–L5 (the last three alcohol fermentation stages) were significantly higher than those detected in the other samples (*p* < 0.05). The titratable acidity of the fermented grains increased significantly from S1 to L1 (*p* < 0.05), peaking at 2.08° at the end. Starch and reducing sugar are two important factors in determining the progress of fermentation. The starch amount gradually reduced through the whole fermentation process, dropped to 16.38% in the end. High reducing sugar contents were detected in the samples from L2–S4 (2.46–2.71%), and thereafter decreased rapidly to 0.88% (L5).

**TABLE 2:** Physiological and chemical properties of fermented grains^a^.

	Stacking fermentation					Alcohol fermentation				
	S1	S2	S3	S4	S5	L1	L2	L3	L4	L5
Moisture (%, m/m)	42.3 ± 0.35d	44.15 ± 0.61c	45.57 ± 0.58bc	46.3 ± 0.99b	48.75 ± 1.38a	44.25 ± 1.15d	45.86 ± 0.75cd	47.17 ± 0.59c	49.95 ± 0.93b	52.5 ± 1.68a
Acidity (°)	0.49 ± 0.07d	1.36 ± 0.16bc	1.28 ± 0.16bc	1.38 ± 0.10b	1.64 ± 0.18a	1.55 ± 0.15bc	1.40 ± 0.19cd	1.43 ± 0.26cd	1.79 ± 0.14ab	2.08 ± 0.12a
Reducing sugar (%, m/m)	1.03 ± 0.06c	1.63 ± 0.37bc	2.66 ± 0.55a	2.71 ± 0.33a	1.88 ± 0.27b	1.27 ± 0.24bc	2.46 ± 0.34a	2.54 ± 0.46a	1.72 ± 0.23b	0.88 ± 0.19c
Starch (%, m/m)	27.38 ± 0.22a	25.71 ± 0.32b	23.29 ± 0.47c	21.12 ± 1.26d	19.11 ± 1.02e	25.65 ± 0.44a	23.39 ± 0.59b	21.05 ± 0.49c	18.5 ± 0.26d	16.38 ± 0.50e
Ethanol (%, m/m)	0.47 ± 0.23cd	0.29 ± 0.05d	0.85 ± 0.09ab	1.13 ± 0.31a	0.76 ± 0.19bc	4.22 ± 0.46c	2.69 ± 0.40c	11.84 ± 0.91b	15.07 ± 1.88a	12.39 ± 1.11b
Temperature (°C)	53.5 ± 2.1a	40.0 ± 1.5d	46.5 ± 1.8b	45.9 ± 1.6bc	43.1 ± 1.2bcd	47.1 ± 2.5a	39.2 ± 1.3cd	42.0 ± 0.7b	41.4 ± 1.7bc	39.1 ± 0.5cd

*^*a*^The minimum significant difference (*p* < 0.05) in the physiochemical properties of stacking and alcohol fermented grains was analyzed by LSD-*t* test, respectively. Means within a column followed by the same letter are not significantly different (*P* > 0.05). The stacking-fermented samples were labeled as S1–S5, while L1–L5 stand for the samples collected during ethanol fermentation.*

### Overall Microbial Diversity Revealed by High-Throughput Sequencing

The deep sequencing of microbial communities originated 4.69 × 10^6^ sequences of V3–V4 and ITS1 regions, of which 4.26 × 10^6^ sequences passed through the quality control screening, representing a 90.8% acceptance rate for high quality sequences (2.21 × 10^6^ reads for V3–V4 and 2.05 × 10^6^ sequences for ITS1). The number of high-quality sequences per sample ranged from 2.56 × 10^4^ to 5.60 × 10^4^. Sequence clustering with a genetic distance of 3% produced 562 OTUs for V3–V4 and 836 OTUs for ITS1. The coverage for the microbial communities was determined by comparing the obtained number of OTUs with the predicted OTU numbers. On average, 90.12 and 85.67% of the prokaryotic and eukaryotic diversities were uncovered, respectively (Tables 3, 4). Alpha-diversity (Shannon indices) was calculated at OTU levels to assess variations in microbial diversity in different fermentation batches. A clear correlation was observed between the microbial diversity and the fermentation stage. In each fermentation batch, a high level of microbial diversity was found in the stacking-fermented grains, and the richness decreased during the alcohol fermentation stage.

**TABLE 3:** Richness and diversity estimations of OTUs derived from 16S rRNA gene sequencing.

Sample	0.03 distance^a^		Coverage^b^	Shannon indices^c^
	OTU obtained	Estimated OTUs		
**Stacking fermentation**				
S1^d^	326.33 ± 10.69	351.84 ± 13.76	92.77 ± 0.91	3.05 ± 0.57b
S2	288.00 ± 10.00	312.54 ± 12.61	92.16 ± 0.67	2.11 ± 0.18c
S3	319.00 ± 3.00	351.35 ± 4.16	90.81 ± 1.90	4.28 ± 0.84a
S4	227.67 ± 5.51	232.78 ± 9.10	97.85 ± 2.26	4.46 ± 0.25a
S5	103.00 ± 5.00	110.52 ± 7.35	93.29 ± 3.04	2.21 ± 0.28bc
**Alcohol fermentation**				
L1	186.00 ± 5.57	206.79 ± 3.69	89.98 ± 3.83	1.28 ± 0.24a
L2	200.67 ± 7.77	265.47 ± 10.35	75.74 ± 5.73	0.40 ± 0.09b
L3	26.67 ± 1.53	31.67 ± 1.53	84.22 ± 2.94	0.04 ± 0.01c
L4	27.33 ± 1.53	29.67 ± 2.52	92.30 ± 3.47	0.07 ± 0.01c
L5	44.33 ± 4.04	48.11 ± 1.65	92.06 ± 6.04	0.30 ± 0.04b

*^*a*^OTUs were determined at a genetic distance of 3%. ^*b*^The coverage was determined as being the ratio between the observed OTUs and estimated OTUs (*Chao1*). ^*c*^The minimum significant difference (*p* < 0.05) in the biodiversity of stacking and alcohol fermented samples was analyzed by ANOVA, respectively. Shannon indices followed by the same letter are not significantly different (*P* > 0.05). ^*d*^The stacking-fermented samples were labeled as S1–S5, while L1–L5 stand for the samples collected during ethanol fermentation.*

**TABLE 4:** Richness and diversity estimations of OTUs derived from ITS region sequencing.

Sample	0.03 distance^a^		Coverage^b^	Shannon indices^c^
	OUT obtained	Estimated OTUs		
**Stacking fermentation**				
S1^d^	426.00 ± 21.28	516.97 ± 18.23	82.55 ± 6.65	3.44 ± 0.71b
S2	536.33 ± 18.58	576.69 ± 14.54	92.99 ± 0.96	5.42 ± 0.42a
S3	369.67 ± 11.24	458.36 ± 18.80	80.76 ± 4.62	2.87 ± 0.19b
S4	24.67 ± 2.89	30.33 ± 2.52	81.82 ± 8.92	0.79 ± 0.23c
S5	26.67 ± 2.52	30.17 ± 2.47	88.46 ± 6.01	0.79 ± 0.26c
**Alcohol fermentation**				
L1	391.00 ± 4.58	452.09 ± 22.71	86.67 ± 5.37	3.33 ± 0.25a
L2	278.33 ± 10.50	323.76 ± 14.34	85.99 ± 0.73	3.07 ± 0.62a
L3	45.33 ± 3.06	51.6 ± 3.86	87.99 ± 5.50	1.59 ± 0.17b
L4	24 ± 1.73	27.67 ± 1.15	86.76 ± 5.58	0.62 ± 0.08c
L5	27.67 ± 2.31	33.5 ± 3.28	82.69 ± 2.97	0.76 ± 0.12c

*^*a*^OTUs were determined at a genetic distance of 3%. ^*b*^The coverage was determined as being the ratio between the observed OTUs and estimated OTUs (*Chao1*). ^*c*^The minimum significant difference (*p* < 0.05) in the biodiversity of stacking and alcohol fermented samples was analyzed by ANOVA, respectively. Shannon indices followed by the same letter are not significantly different (*P* > 0.05). ^*d*^The stacking-fermented samples were labeled as S1–S5, while L1–L5 stand for the samples collected during ethanol fermentation.*

### Dynamics of Bacterial Communities

**FIGURE 1:**
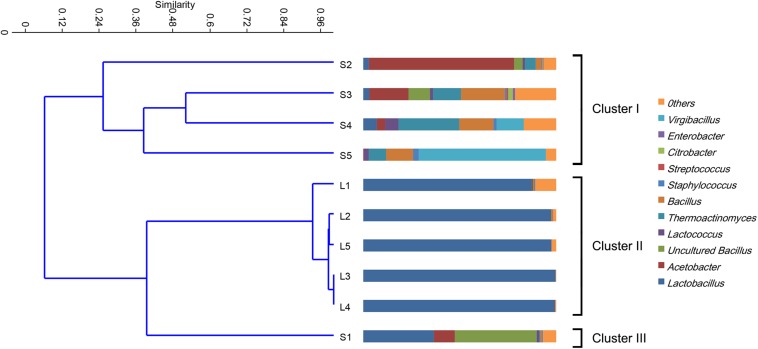
Clustering and relative abundance of bacterial taxa across samples. Samples were clustered by unweighted pair group method with arithmetic mean (UPGMA). The scale bar indicates the distance among different samples in UniFrac units, where 0 stands for total dissimilarity and 1 stands for being completely identical. The prokaryotic genera of rare population (<0.5%) were placed in an artificial group designed as “others.”

The bacterial communities in the fermented grains were mainly characterized by a high percentage of microorganisms from *Bacilli* and α*-proteobacteria* classes. Microorganisms, namely, γ*-proteobacteria* and *Halobacterium*, were identified at lower abundance. At the genus level, the bacterial communities were mostly characterized by *Lactobacillus*, *Acetobacter*, *Bacillus*, *Virgibacillus*, and *Thermoactinomyces*. The relative abundance of these dominant genera varied in both fermentation stages and fermentation batches (Figure 1). In the stacking fermentation process, approximately 49.8% (on average) of the sequences belonged to *Lactobacillus* and *Acetobacter* in the former three fermentation batches. This amount decreased to 11.5% during the fourth fermentation batch and almost disappeared at the end of the fermentation. Conversely, the percentage of sequences belonging to *Bacillus* and *Virgibacillus* increased from 31.7 to 79.9% in the last three batches. In the alcohol fermentation stages, the compositions of bacterial populations were highly similar at the genus level. About 95% of the 16S rRNA gene sequences could be assigned to the genus *Lactobacillus*. When the unweighted pair group method with arithmetic mean (UPGMA) clustering was conducted to evaluate the similarities in different communities, the bacterial communities were grouped into three clusters according to the percentage of specific bacteria (Figure 1). The bacterial communities in S1 formed one cluster (Cluster III). Other bacterial communities were grouped according to their fermentation stage. Cluster I grouped the samples S2–S5, whereas cluster II contained L1–L5, which were collected from the alcohol fermentation stages. Significant differences (*R*_ANOSIM_ = 0.43, *p* = 0.1%) were observed between cluster I and II samples. The bacterial communities in cluster I were mainly characterized by *Thermoactinomyces*, *Lactococcus*, and *Bacillus*, which contributed 69% to the group similarity. The average similarity in cluster II comprised mainly of contributions from *Lactobacillus.*

### Diversity of Fungi

**FIGURE 2:**
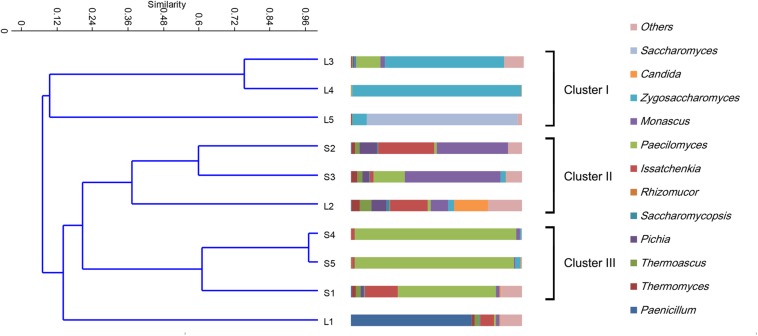
Clustering and relative abundance of fungal taxa across samples. Samples were clustered by unweighted pair group method with arithmetic mean (UPGMA). The fungal members of rare population (<0.5%) were placed in an artificial group designed as “others.”

The fungal taxonomic distribution at the genus level is shown in Figure 2. In the stacking fermentation stages, approximately 47.9–96.9% of the total sequences could be assigned to four genera (*Paecilomyces*, *Thermomyces*, *Thermoascus*, and *Monascus*). The most abundant group was *Paecilomyces*, followed by *Thermomyces* and *Thermoascus*. Large percentages of *Monascus* (41.7–56.0%) were only detected in the S2 and S3 fermentation stages. The percentage of *Paecilomyces* decreased sharply in the S2 samples (the second stacking fermentation stage), increased in the S3 and eventually dominated the population. In the alcohol fermentation stages, the yeast population increased in most samples. *Zygosaccharomyces* and *Saccharomyces* accounted for more than 95% of the total abundance in the fourth and fifth fermentation batches. UPGMA clustering indicated that the fungal communities could be mainly divided into four categories (Figure 2). The first group includes samples L3–L5. Group II consisted of samples S2, L2, and S3. Group III comprises S1, S4, and S5. The fungal communities in L1 were distinct from those of other samples and cannot be clustered into any group. In general, the fungal community was highly dynamic and presented more complex alterations compared with bacterial communities.

### Microbial Composition in Relation to Environmental Variables

**FIGURE 3:**
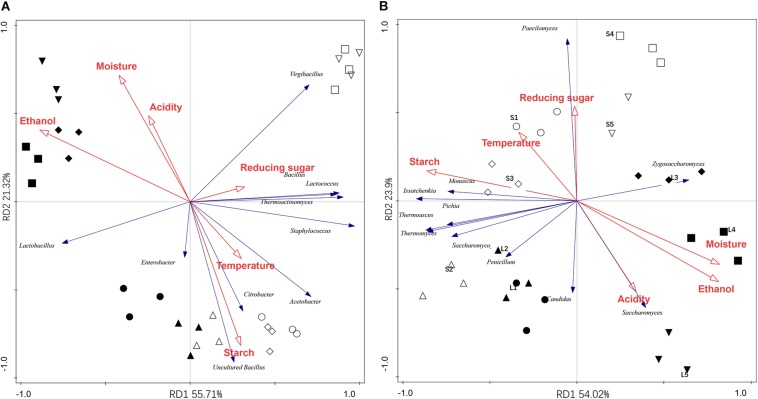
Redundancy discriminate analysis (RDA) for the dominant bacteria **(A)** and fungi presented **(B)** in different fermentation batches. Circle (◯/⚫), triangle (△/▲), diamond (♢/◆), square (□/■), and inverted triangle (▽/▼) represented the microbial communities presented in the first to the fifth fermentation batches. Hollow and solid represented the samples from stacking and alcohol fermentation stages.

The relationship between the microbial community and environmental variables were illustrated by RDA analysis. As shown in Figure 3A, the first and second axes explained 55.7 and 21.3% of the total bacterial variability. Axis 1 separates microbial communities of the stacking fermentation stage from those of the baijiu fermentation stages, and the samples from different batches were separated by axis 2. The significant factors affecting the bacterial community were ethanol (explaining 47.6% of the variation), starch (19.2%), water (6.3%), and reducing sugar (4.8%) (Table 5). Increases in the percentage of bacterial genera along axis 1 (such as *Bacillus, Lactococcus*, *Thermoactinomyces*, *Staphylococcus*, *Acetobacter*, and *Citrobacter*) were positively correlated with the contents of starch and reducing sugar. However, increases in the percentage of *Lactobacillus* were positively associated with ethanol and water contents. The interactions between environmental variables and fungal community composition are shown in Figure 3B. The RDA explained 89.5% of the variation in the fungal community data, of which 77.9% were explained by the first two axes. The fungal communities in different samples were also clearly separated. The fungal communities were significantly affected by ethanol content (28.9%), followed by starch (27.0%), water (27.0%), and acidity (12.3%), all of which explained 95.2% of the variation (Table 5). Increases in the percentages of *Saccharomyces* and *Zygosaccharomyces* along axis 1 were positively correlated with acidity, alcohol and water content. Fungal species loaded negatively on axis 1 were positively associated with the starch content. Increases in *Paecilomyces* along axis 2 were positively correlated with reducing sugar. The data above indicated that high ethanol and water content, as well as the acidic fermentation environment could facilitate the growth of *Lactobacillus*, *Saccharomyces*, and *Zygosaccharomyces* in the last alcohol fermentation stages.

**TABLE 5:** Explanation of the fermentation parameters used in RDA with significant correlation to microbial or fungal communities.

	Explanation of the factors (%)^a^	
Fermentation parameters	Bacterial community	Fungal community
Ethanol	47.6**	28.9**
Starch	19.2**	27.0**
Moisture	6.3*	27.0**
Acidity	–^b^	12.3*
Sugar	4.8*	12.1*
Temperature	–	–

^*a*^Asterisks indicated the factor significantly influenced the microbial community among the samples using Monte Carlo permutational tests (**p < 0.01, *p < 0.05). ^*b*^Dashes means the factor did not significantly influenced the microbial community among the samples.

### Association Between Volatile Compounds and Microbial Community

**FIGURE 4:**
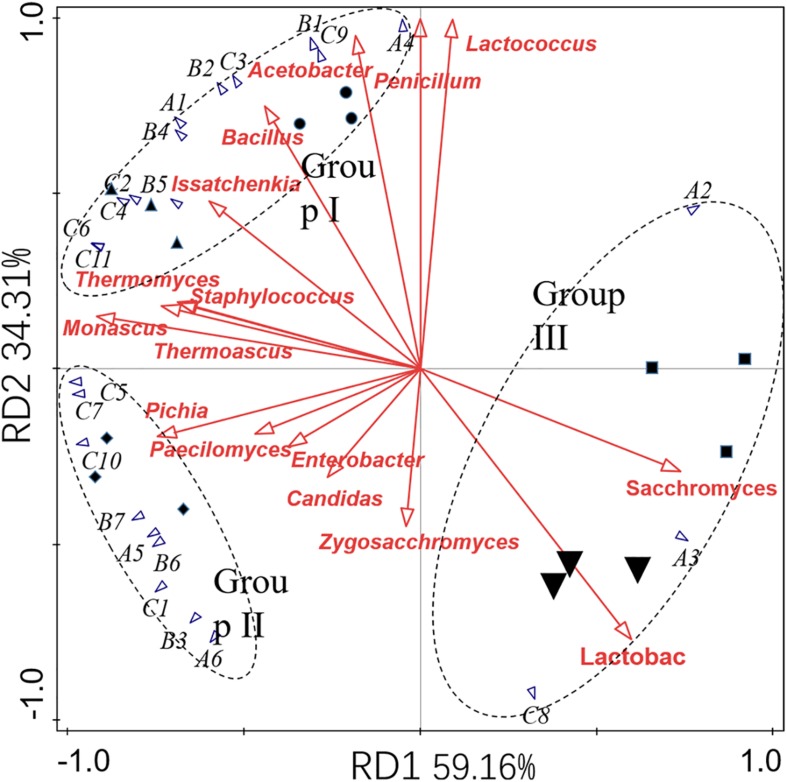
RDA analysis of the microbial communities and volatile profiles presented in fermented grains. Circle (⚫), triangle (▲), diamond (◆), square (◆), and inverted triangle (▼) represented the samples collected from the first to the fifth fermentation batches. The codes A1 to C11 represent the volatile compounds listed in Table 6.

GC-MS was employed to analyze volatile compounds in each fermentation sample. Twenty-two volatiles were identified based on their mass spectra, including 6 esters, 7 acids, and 11 alcohols (Table 6). RDA analysis (Figure 4) revealed that these volatiles can be divided into three groups based on their loading on the axes. Group I containing 12 volatile compounds. Most of these compounds were higher alcohols and acids, which were presented in high concentrations in the fermented grains sampled in the former two batches and had a positive association with *Issatchenkia*, *Penicillium*, *Acetobacter*, *Bacillus*, and *Lactococcus*. Nine compounds were categorized into group II, including three acids (caproic acid, methyl propionic acid, and pentanoic acid), two esters (ethyl caprylate and ethyl oenanthate), and three alcohols (hexanol, pentanol, and apentanol). High concentrations of these compounds were characteristic of the samples collected in the third fermentation batch, where high percentages of *Thermomyces*, *Thermoascus*, *Monascus*, and *Pichia* were observed. The fermented grains sampled from the fourth and fifth fermentation batches located near each other, meaning that they had similar volatile profiles. High concentrations of ethyl lactate, 2,3-butanediol and ethyl caproate were detected. Compared with the samples collected in the former three batches, lower concentrations of acids and alcohols were observed.

**TABLE 6:** Concentration of the volatile compounds detected in the fermented grains.

Code	Compound	Concentration (mg/L)				
		A	B	C	D	E
A1	Ethyl acetate	342.15 ± 30.69	242.91 ± 18.23	135.40 ± 10.46	73.37 ± 6.77	40.84 ± 7.53
A2	Ethyl lactate	119.62 ± 5.45	74.54 ± 4.53	85.80 ± 7.76	152.65 ± 11.08	115.95 ± 13.92
A3	Ethyl caproate	5.64 ± 1.53	6.90 ± 0.92	6.90 ± 0.76	9.37 ± 2.53	10.57 ± 1.77
A4	Ethyl valerate	4.14 ± 0.49	1.64 ± 0.46	0.83 ± 0.33	1.04 ± 0.38	1.66 ± 0.31
A5	Ethyl oenanthate	0.35 ± 0.11	0.26 ± 0.06	0.14 ± 0.09	0.12 ± 0.08	0.19 ± 0.08
A6	Ethyl caprylate	0.06 ± 0.02	3.60 ± 0.46	0.68 ± 0.15	0.33 ± 0.16	0.21 ± 0.04
B1	Acetic acid	161.16 ± 21.81	78.81 ± 11.83	44.03 ± 6.61	41.42 ± 8.65	45.44 ± 3.94
B2	Propionic acid	64.18 ± 3.69	49.34 ± 2.23	37.80 ± 2.77	29.22 ± 4.84	25.80 ± 1.46
B3	Caproic acid	7.81 ± 0.83	6.18 ± 2.15	8.04 ± 1.36	8.86 ± 1.38	1.66 ± 0.65
B4	Butyric acid	21.67 ± 3.78	27.60 ± 3.23	12.30 ± 3.46	12.28 ± 3.54	8.41 ± 2.96
B5	3-methylbutyric acid	1.39 ± 0.76	1.48 ± 0.61	1.07 ± 0.38	0.94 ± 0.30	1.41 ± 0.07
B6	Pentanoic acid	1.95 ± 0.30	2.74 ± 0.92	1.32 ± 0.23	1.17 ± 0.31	0.28 ± 0.08
B7	2-methylpropionic acid	3.25 ± 0.23	0.48 ± 0.06	2.02 ± 0.15	0.20 ± 0.08	1.40 ± 0.38
C1	Methanol	5.20 ± 0.51	43.49 ± 8.93	13.57 ± 0.61	10.80 ± 0.84	15.26 ± 3.22
C2	1-methylpropanol	36.84 ± 3.84	45.95 ± 4.61	5.08 ± 0.83	3.15 ± 1.61	4.40 ± 1.69
C3	1-propanol	172.58 ± 9.85	100.51 ± 9.83	49.56 ± 11.94	44.97 ± 4.53	61.28 ± 5.23
C4	2-methyl-1-propanol	20.24 ± 6.77	20.72 ± 1.46	14.18 ± 2.83	7.18 ± 0.89	11.06 ± 1.69
C5	2-pentanol	0.57 ± 0.38	1.38 ± 0.53	0.39 ± 0.12	0.22 ± 0.08	0.49 ± 0.21
C6	1-butanol	12.83 ± 1.80	18.71 ± 3.92	4.31 ± 1.84	2.70 ± 0.15	5.08 ± 0.83
C7	1-hexanol	2.71 ± 0.23	6.22 ± 2.76	1.91 ± 0.76	1.02 ± 0.31	2.49 ± 0.37
C8	2,3-butanediol	2.87 ± 0.76	1.83 ± 0.23	7.80 ± 0.23	12.77 ± 3.74	4.93 ± 0.84
C9	1,2-propanediol	4.73 ± 0.33	3.43 ± 0.30	2.16 ± 0.37	3.06 ± 0.69	3.29 ± 0.72
C10	1-pentanol	3.54 ± 0.61	6.66 ± 0.84	1.90 ± 0.83	2.96 ± 0.71	3.70 ± 1.76
C11	3-methyl-1-butanol	35.38 ± 4.87	50.93 ± 2.92	28.92 ± 6.66	16.93 ± 5.15	17.52 ± 3.54

## Discussion

Evaluating microbial succession and their association with environmental factors and volatile profiles during Chinese baijiu fermentation could identify the major microbes associated with baijiu production. Moreover, this is important for the effective management of the fermentation process. In this study, 48 h-fermented samples were selected for analysis of the microbial community during stacking fermentation, at which the temperature of the stacking-fermented grains begins to increase (Li et al., 2016). Analysis of microbial community at this time point can better represent the microorganisms that initiated the stacking fermentation. In baijiu fermentation stages, our previous data revealed that the numbers of yeast and bacteria reached their plateau at 15 days (Liu, 2015). Similar variation trend was also observed by Kong et al. (2014), indicating the formation of mature microbiota.

### Microbial Dynamics and Their Correlation With Environmental Variables

Significant differences in the microbial community structure were observed between different fermentation batches. In the former three fermentation batches, large percentages of *Thermomyces*, *Thermoascus*, *Monascus*, *Bacillus*, and *Acetobacter* were identified in the stacking fermentation stages. In the alcohol fermentation stages, large percentages of *Issatchenkia*, *Pichia*, and *Lactobacillus* were observed. In contrast, *Bacillus*, *Virgibacillus*, and *Paecilomyces* became the dominant microorganisms in the stacking fermentation stages of the last two fermentation batches (S4–S5). Meanwhile, *Lactobacillus*, *Saccharomyces*, and *Zygosaccharomyces* dominated the microbial community in the alcohol fermentation stages (L4–L5). Considering that Chinese baijiu is produced by solid-state fermentation, the biological heat generated by microbial activity is difficult to spread out into surroundings, the temperature of fermented grains can reach 60°C. Almost all the microorganisms identified in this study had high levels of heat resistance. Therefore, temperature cannot be the deciding environmental factor that influences the formation of functional microbiota. In the former three fermentation batches, high concentrations of reducing sugar were detected. *Thermomyces* and *Thermoascus* are two famous fungi that colonized at high temperature and produced many kinds of thermos-tolerant enzymes to help the degradation of plant materials (Jensen et al., 2002; Kalogeris et al., 2003; McClendon et al., 2012; Winger et al., 2014). *Bacillus* species are ubiquitous spore-forming bacteria. Many *Bacillus* species also secrete a wide range of hydrolytic enzymes that are important in sorghum hydrolysis and facilitates starch liquefaction and saccharification (Xiong et al., 2014). The high concentrations of reducing sugar generated by starch degradation may in turn promote the growth of other microorganisms, leading to high microbial diversity. As the production proceeds, in the fourth and fifth fermentation batches, high concentrations of water and ethanol as well as high acidity were detected. The microbial diversity decreased sharply. Many microorganisms might not endure the elevated levels of alcohols and/or acids in the last two fermentation batches. *Lactobacillus* is a genus of Gram-positive, aerotolerant or microaerophilic, non-spore-forming bateria (Stefanovic et al., 2017). In the last two fermentation batches, the high moisture content could help remove oxygen from the fermented grains, thereby creating a good environment that faciliated the growth of *Lactobacillus*. In addition, many *Lactobacillus* species shows ethanol-tolerant ability, were previously used or identified in alcoholic beverages fermentation (Bravo-Ferrada et al., 2013; Bartle et al., 2019). *Sacchraomyces* and *Zygosaccharomyces* are the dominant yeasts detected in the last fermentation batch. *Saccharomyces*, especially *S. cerevisiae*, is the most effective ethanol producer so far and is the most common yeast in indigenous fermented foods and alcoholic beverages. *Zygosaccharomyces* could adapt to high-acid conditions, and have demonstrated to play an important role in Chinese sauce-flavor baijiu-making process (Wu et al., 2012).

### Association Between Microbial Community and Volatile Profiles

Esters, alcohols, and acids are important volatile compounds in Chinese baijiu, and their composition and concentration greatly influenced the quality of the product. Esters are essential ingredients that yield fruity and floral flavors, especially ethyl hexanoate, which is recognized as a key compound with high oslae value in Chinese miscellaneous-flavor and strong-flavor baijiu (Chen et al., 2013). Yeasts have generally been recognized as main ester-producers (Wu et al., 2012). *Monascus* secretes a large amount of esterase (Leng and Xu, 2015), the high percentage of this species in the former three batches may also promote the formation of esters. The influence of higher alcohols to the sensory characteristics of baijiu is closely related to their concentration. A proper concentration of higher alcohols will provide baijiu with harmonious taste and increase its sweetness and aftertaste (Zhang et al., 2009). However, high concentrations of high alcohols generate a fuel-like flavor. Many higher alcohols identified in the present study can be produced from branched-chain amino acids through the catabolic pathway (Vidal et al., 2013). In the former three fermentation batches, sorghum was greatly degrade with the growth of *Thermomyces*, *Thermoascus*, and *Bacillus*. The released amino acids may greatly enhance the generation of higher alcohols. The concentrations of acids in this study were positively correlated with the presence of *Issatchenkia*. This phenomenon is supported by the data obtained by Wu et al. (2012), who reported that *I. orientalis* produced the largest quantity and richest variety of volatile acids during Chinese sauce-flavor baijiu fermentation. Compared with volatiles generated in the former three batches, the samples collected from the last two batches were characterized by high concentrations of ethyl lactate, 2,3-butanediol and ethyl caproate. The concentrations of other volatiles are relatively low. This finding may explain why sensory analysis revealed that the baijiu distilled from the latter two batches has higher quality (Chen et al., 2013; Fresno et al., 2017). Compared with the microbial community in the former three batches, the composition of microorganisms identified in the latter two batches is rather simple. *Saccharomyces*, *Zygosaccharomyces*, and *Lactobacillus* dominant the baijiu fermentation stages. Similar to many other alcoholic beverages, such as wine and sour beer (Bartle et al., 2019; Dysvik et al., 2019), the production of miscellaneous baijiu seems also depend on the co-fermentation of lactic acid bacteria and yeasts. During wine fermentation, co-inoculation of lactic acid bacteria with yeast not only reduces the total acidity but also increases the ripe fruits notes associated with esters (Bravo-Ferrada et al., 2013). The buttery and creamy aromas linked to diethyl succinate and ethyl lactate are also enhanced (Tristezza et al., 2016). To our best knowledge, the interaction between *Lactobacillus* and yeasts during Chinese baijiu fermentation remains largely uncovered. Isolation of yeast-compatible *Lactobacillus* may facilitate its biotechnological uses in the baijiu industry and contribute to the modernization of the traditional fermentation skills.

In summary, this study revealed the microbial diversity and structure in the fermentation of Baiyunbian baijiu. Based on GC-MS analysis, high quality baijiu produced from the last two fermentation batches was characterized by high concentrations of ethyl lactate, 2,3-butanediol and ethyl caproate. The concentrations of higher alcohols and acids were relatively low. *Paecilomyces*, *Bacillus*, and *Virgibacillus* were identified as key microbes in the stacking fermentation stages of these two batches, while *Lactobacillus*, *Saccharomyces*, and *Zygosaccharomyces* dominated the alcohol fermentation stages. High acidity, large contents of moisture and ethanol play important roles in promoting the formation of functional microbiota. The result expand our understanding on the factors that influenced the baijiu quality. With these data, the quality of baijiu can be stabilized by control of fermentation conditions and adjustment of microbial community.

## Data Availability Statement

The RNA-seq datasets for this study can be found in the NCBI short read archive database under the BioProject accession number PRJNA379892.

## Author Contributions

LM conceived the experiment. PL performed the experiment and wrote the manuscript.

## Conflict of Interest

The authors declare that the research was conducted in the absence of any commercial or financial relationships that could be construed as a potential conflict of interest.
